# Mesenchymal stem cells in synovial fluid increase in number in response to synovitis and display more tissue-reparative phenotypes in osteoarthritis

**DOI:** 10.1186/s13287-023-03487-1

**Published:** 2023-09-08

**Authors:** Hideto Furuoka, Kentaro Endo, Ichiro Sekiya

**Affiliations:** Center for Stem Cell and Regenerative Medicine, Tokyo Medical and Dental University (TMDU), 1-5-45 Yushima, Bunkyo-Ku, Tokyo, 113-8510 Japan

**Keywords:** Synovial fluid, Osteoarthritis, Mesenchymal stem cell, Synovitis, Articular cartilage, Meniscus

## Abstract

**Background:**

Synovial fluid mesenchymal stem cells (SF-MSCs) originate in the synovium and contribute to the endogenous repair of damaged intra-articular tissues. Here, we clarified the relationship between their numbers and joint structural changes during osteoarthritis (OA) progression and investigated whether SF-MSCs had phenotypes favorable for tissue repair, even in an OA environment.

**Methods:**

Partial medial meniscectomy (pMx) and sham surgery were performed on both knees of rats. SF and knee joints were collected from intact rats and from rats at 2, 4, and 6 weeks after surgery. SF was cultured for 1 week to calculate the numbers of colony-forming cells and colony areas. Joint structural changes were evaluated histologically to investigate their correlation with the numbers and areas of colonies. RNA sequencing was performed for SF-MSCs from intact knees and knees 4 weeks after the pMx and sham surgery.

**Results:**

Colony-forming cell numbers and colony areas were greater in the pMx group than in the intact and sham groups and peaked at 2 and 4 weeks, respectively. Synovitis scores showed the strongest correlation with colony numbers (*R* = 0.583) and areas (*R* = 0.456). RNA sequencing revealed higher expression of genes related to extracellular matrix binding, TGF-β signaling, and superoxide dismutase activity in SF-MSCs in the pMx group than in the sham group.

**Conclusion:**

The number of SF-MSCs was most closely correlated with the severity of synovitis in this rat OA model. Tissue-reparative gene expression patterns were observed in SF-MSCs from OA knees, but not from knees without intra-articular tissue damage.

**Supplementary Information:**

The online version contains supplementary material available at 10.1186/s13287-023-03487-1.

## Background

Osteoarthritis (OA) of the knee, which affects approximately 250 million people worldwide, is a very serious and increasing cause of disability [1]. OA is characterized by progressive cartilage destruction [2], meniscus degeneration [3], subchondral bone sclerosis [4], and synovitis [5]. Some treatment options can reduce OA pain, but no cures or disease-modifying drugs are presently available. Therefore, many researchers have devoted significant effort toward developing new OA treatments.

One of the most attractive OA solutions is the intra-articular injection of mesenchymal stem cells (MSCs). MSCs have self-renewal and chondrogenic abilities and secrete abundant immunomodulatory/trophic factors in vivo [6]. These cells reside in various tissues, but synovial MSCs are the most promising cell types for cartilage regeneration because they are committed to a chondrogenic lineage [7]. We have previously confirmed that the intra-articular injection of autologous synovial MSCs into the knees of patients with OA can improve their pain scores and the volume of articular cartilage [8].

MSCs exist in the synovium, but they are also present in synovial fluid (SF) [9]. Although synovial fluid MSCs (SF-MSCs) are seldom observed in healthy knees [10], their numbers increase in OA knees and in knees with injuries to the meniscus or anterior cruciate ligament [11–14]. We previously reported the in vitro release of SF-MSCs from OA synovium and that the released SF-MSCs have gene expression profiles and therapeutic potentials comparable to those of synovial MSCs [14–17]. Baboolal et al. also showed that SF-MSCs immediately adhered to the surface of degenerated cartilage following intra-articular injection in a canine OA model [18]. These findings suggest that SF-MSCs naturally originate in the synovium and contribute to the endogenous repair of damaged intra-articular tissues [19]. A better understanding of how endogenous SF-MSCs are mobilized could therefore lead to the development of novel OA therapies that would not require the injection of exogenous MSCs.

A previous study revealed changes in the number and gene expression signature of SF-MSCs from OA patients before and after knee joint distraction surgery [17]. However, the mechanism by which the numbers of SF-MSCs change with OA progression and the nature of the structural changes of the joint that are predominantly associated with the changes in SF-MSC numbers remain unclear. Another unanswered question is whether endogenous SF-MSCs undergo tissue destructive or reparative phenotype changes in response to the inflammatory and catabolic environment associated with OA.

In this study, we examined the relationship between time-course changes in the numbers of SF-MSCs and the histological changes in knee joint tissues in a rat OA model. We also investigated whether the gene expression profiles of SF-MSCs from rat OA knees were favorable for endogenous cartilage repair.

## Methods

### Animal surgery

A total of 45 wild-type male Lewis rats (Sankyo Labo Service Corporation, Tokyo, Japan) at 10–12 weeks old were used for the experiments (mean weight 275 ± 18 g). All animal care and experiments were conducted in accordance with the institutional guidelines of the Animal Committee of Tokyo Medical and Dental University (permission number: A2021-267A) and the ARRIVE guidelines. Partial medial meniscectomy (pMx) was performed on either the right or left knee under 2% isoflurane anesthesia [20]. For pMx surgery, the meniscotibial ligament was transected and the anterior half of medial meniscus was removed. The joint capsule and skin were closed with 5–0 nylon sutures. The contralateral knee received sham surgery (joint capsule incision). At 2, 4, and 6 weeks after the surgery, the rats were euthanized using CO_2_. After confirming respiratory arrest and loss of consciousness, the knee joints and SF were harvested. Intact rats that received no surgery were also prepared. All rats were allowed to walk freely in a cage. The rats were kept in approved animal-care facilities and were housed 2 per cage in 280 × 440 × 205 mm (L × W × H) cages (TP-105, TOYO-LABO, Tokyo, Japan). All rats were provided with a certified rat diet (Rodent Diet CE-2, CLEA Japan, Tokyo, Japan) and water ad libitum. We used 21 rats for the evaluation of colonies and joint tissues, 8 rats for in vitro characterization of colony-forming cells, 4 rats for flow cytometry analysis of synovial tissue, and 12 rats for RNA sequencing.

### Culture of colony-forming cells in SF

SF was collected from rat knee joints by pumping the joints twice with 50 µL of phosphate-buffered saline (PBS) [21]. The collected fluid was plated in a 145 cm^2^ dish (Thermo Fisher Scientific, Waltham, MA, USA) and cultured in growth medium consisting of α-modified essential medium (α-MEM; Thermo Fisher Scientific), 10% fetal bovine serum (FBS; Thermo Fisher Scientific), and 1% antibiotic–antimycotic (Thermo Fisher Scientific) at 37 °C in 5% humidified CO_2_. Seven days after the initial plating, the cells were stained with 0.5% crystal violet (Wako, Osaka, Japan). The numbers and sizes of the colonies were assessed using Fiji/Image J (National Institute of Health, Bethesda, MD, USA) and the “Analyze Particles” command [22]. Colonies less than 1 mm^2^ in area were ignored. The MSC properties were evaluated by detaching colony-forming cells from intact knees and from knees 4 weeks after the sham and pMx surgeries with 0.25% trypsin (Thermo Fisher Scientific) and expanding the detached MSCs in growth medium to passage 2.

### Histology

Rat knee joints were fixed with 10% formalin neutral buffer solution (Wako) for 7 days at room temperature, decalcified in 20% ethylenediaminetetraacetic acid (EDTA) for 21 days, and then embedded in paraffin wax. The specimens were cut into 5 µm sagittal sections and stained with hematoxylin/eosin (HE) and safranin O/fast green. Synovitis, cartilage degeneration, meniscus regeneration, and bone marrow lesions (BMLs) were evaluated using Krenn’s score [5] (Additional file 2: Table S1), the OARSI score [2] (Additional file 2: Table S2), the modified Pauli’s score [3, 23] (Additional file 2: Table S3), and the osteoarthritis bone score (OABS) [4] (Additional file 2: Table S4), respectively. Infrapatellar fat pad (IFP) fibrosis was assessed as the percentage of the fibrotic area determined using Fiji/ImageJ.

### Immunohistochemistry

Immunostaining of CD68, CD73, and ZO-1 was conducted by immersing the sections in 10 mM Tris containing 1 mM EDTA (pH 9.0) at 60 °C for 16 h to retrieve antigens. The slides were then immersed in methanol containing 0.3% H_2_O_2_ for 30 min and washed with Tris-buffered saline containing 0.1% Tween-20 (TBS-T). After blocking with Blocking One Histo (NACALAI TESQUE, Kyoto, Japan), the sections were incubated overnight at 4 °C with antibody against CD68 (1:200; Novus Biologicals, Littleton, CO, USA), CD73 (1:200; R&D), CD80 (1:100; Bioss Antibodies, Woburn, MA, USA), CD206 (1:500; Abcam), or ZO-1 (1:50; Thermo Fisher Scientific). After three washes with TBS-T, the sections were incubated with secondary antibodies conjugated with horseradish peroxidase (1:200; Abcam) for 1 h at room temperature. DAB solution (Dako) was then applied for 5 min, and the cells were counterstained with hematoxylin. We selected CD68 and CD73 for evaluation because these are general markers for macrophages and MSCs, respectively [24, 25]. ZO-1 is a tight junction protein located on synovial surface and regulates cell migration between the synovium and SF [26]. DAB-positive areas were quantified using the Color Deconvolution plugin for Fiji/Image J in two fields of view of the synovium (465 × 620 µm) for CD68 and CD73, and four fields of view at the surface of the synovium (100 × 500 µm) for ZO-1. After unmixing the hematoxylin and DAB colors, we determined the DAB-positive area using thresholding binary images. All images were acquired under the same light settings.

For immunofluorescence, the sections were incubated overnight at 4 °C with primary antibodies against CD44 (1:200; Proteintech, Rosemont, IL, USA), CD271 (1:200; Millipore, Billerica, MA, USA), and CD73 (1:200; R&D). After three washes with TBS-T, the sections were incubated at room temperature for 1 h with secondary antibodies conjugated with Alexa488 (1:200; Abcam) and Alexa555 (1:200; Abcam). The nuclei were stained with 4′6-diaminido-2-phenylindole (DAPI; Dojindo, Kumamoto, Japan) for 5 min. All images were captured using a BZ-X700 fluorescence microscope (Keyence, Osaka, Japan).

### Surface epitopes

Colony-forming cells from SF were detached at passage 2 with TrypLE (Thermo Fisher Scientific) and suspended in FACS buffer (2% FBS and 5 mM EDTA in PBS). The cells were stained with the CD34-PerCP (Novus), CD44-PE (R&D Systems, Minneapolis, MN, USA), CD45-FITC (BD Biosciences, San Jose, CA, USA), CD90-PE-Cy7 (BD), CD105-APC (Novus), and Ghost Dye Violet 510 for dead cells (Tonbo Biosciences, CA, USA). For CD73, a mouse anti-CD73 antibody (BD) was used, followed by the secondary antibody conjugated with Alexa Fluor 488 (Abcam).

The synovial tissue was analyzed by mincing the synovium and digesting it for 3.5 h at 37 °C with 0.4 mg/mL Liberase (Roche Diagnostics, Mannheim, Germany) and 0.1 mg/mL DNase (Sigma-Aldrich, St Louis, MO, USA). Residual red blood cells were lysed with ACK Lysing Buffer (Thermo Fisher Scientific), the cells were passed through a 70 μm strainer (Greiner Bio-One GmbH, Frickenhausen, Germany) to yield single-cell suspensions. The cells were then stained with the CD44-PE, CD73-PE-Cy7 (Bioss), and CD90-APC (BD), as well as with Ghost Dye Violet 450 (Tonbo Biosciences) to identify dead cells. The proportion of antigen-positive cells was evaluated using a FACSVerse instrument (BD).

### Colony-forming unit (CFU) assays

Colony-forming cells at passage 2 were plated at 100 cells/dish in 60 cm^2^ dishes (Thermo Fisher Scientific) and cultured in growth medium for 2 weeks. The cells were then fixed with 10% neutral buffered formalin and stained with 0.5% crystal violet. Colonies were counted manually, and colonies less than 1 mm^2^ in area were ignored.

### Differentiation assays

Adipogenesis was evaluated by plating 100 cells in a 60 cm^2^ dish and culturing them in growth medium for 14 days to allow colony formation. The medium was then switched to an adipogenic induction medium consisting of growth medium supplemented with 100 nM dexamethasone, 0.5 mM isobutyl methylxanthine (Sigma-Aldrich), 4.5 mg/mL D-( +)-glucose (Wako), and 100 µM indomethacin (Sigma-Aldrich). After 2 weeks of adipogenic induction, the cells were stained with oil red O (Sigma-Aldrich).

Calcification was examined by plating 100 cells in a 60 cm^2^ dish and culturing them in growth medium for 14 days to allow colony formation. Adherent cells were subsequently cultured in an osteogenic induction medium consisting of growth medium supplemented with 50 μg/mL ascorbic acid 2-phosphate (Wako), 100 nM dexamethasone (Wako), and 10 mM β-glycerophosphate (Sigma-Aldrich) After 3 weeks of osteogenic induction, the cells were stained with alizarin red (Sigma-Aldrich).

For chondrogenesis, 2.5 × 10^5^ cells were transferred into a 15 mL tube (Thermo Fisher Scientific) and centrifuged for 10 min at 580 × g. The cell pellets were cultured in chondrogenic induction medium consisting of high glucose Dulbecco’s Modified Eagle Medium (Thermo Fisher Scientific), 1% insulin–transferrin–selenium (ITS; BD), 50 µg/mL ascorbate-2-phosphate, 40 µg/mL L-proline (Sigma-Aldrich), 100 nM dexamethasone, 100 µg/mL pyruvate (Sigma-Aldrich), 1% antibiotic–antimycotic, 10 ng/mL human transforming growth factor-β3 (Miltenyi Biotec Japan, Tokyo, Japan), and 1000 ng/mL human bone morphogenetic protein 2 (Medtronic, Minneapolis, MN). After 3 weeks, the cell pellets were fixed, embedded in paraffin, and sectioned. The slides were stained with safranin O/fast green (Wako).

### RNA sequencing

SF from intact knees and knees 4 weeks after sham and pMx surgery was plated in 145 cm^2^ dishes and cultured in growth medium. After 1 week of cultivation, total RNA was extracted using the RNeasy Mini Kit (QIAGEN, Hilden, Germany). RNA sequencing was performed by the Beijing Genomics Institute (BGI; Shenzhen, Guangdong, China). Briefly, mRNA was isolated using oligo-dT beads and fragmented. Single-strand cDNA was generated using random N6-primed reverse transcription, followed by a second-strand cDNA with dUTP. After PCR amplification, the products were sequenced on the DNBSEQ platform. The sequence reads were aligned to a rat reference genome (GCF_000001895.5_Rnor_6.0).

### Analysis of sequencing data

All data were analyzed using Dr. Tom, a BGI data mining system. The gene expression levels were calculated as transcripts per million (TPM) values. Differentially expressed genes (DEGs) were determined between each group (FDR < 0.05). Gene ontology (GO) enrichment analysis was performed on DEGs that were upregulated in the pMx group compared with the intact and sham groups.

### Statistical analysis

All data were expressed as medians and interquartile ranges (IQRs). All statistical analyses, except for RNA sequencing statistics, were performed using EZR (Saitama Medical Center, Jichi Medical University, Japan), a graphical user interface of R (The R Foundation for Statistical Computing, Vienna, Austria, version 2.7.1) [27]. Statistical significance was accepted at *p* < 0.05. Our null hypotheses were that the histological scores of the sham or pMx groups would not differ from the scores of the intact group at any time point and that the scores of the sham group would not differ from the scores of the pMx group at any time point. The Kruskal–Wallis test with the post hoc Steel test was used for comparisons of time-course changes, and the Mann–Whitney U test was used for comparisons between 2 groups. Spearman’s rank correlation coefficient was used for evaluating correlations. Bonferroni correction was used to adjust the p values of multiple Spearman’s rank correlation coefficients.

## Results

### Changes in the numbers of colony-forming cells in SF and their colony areas

**Fig. 1:**
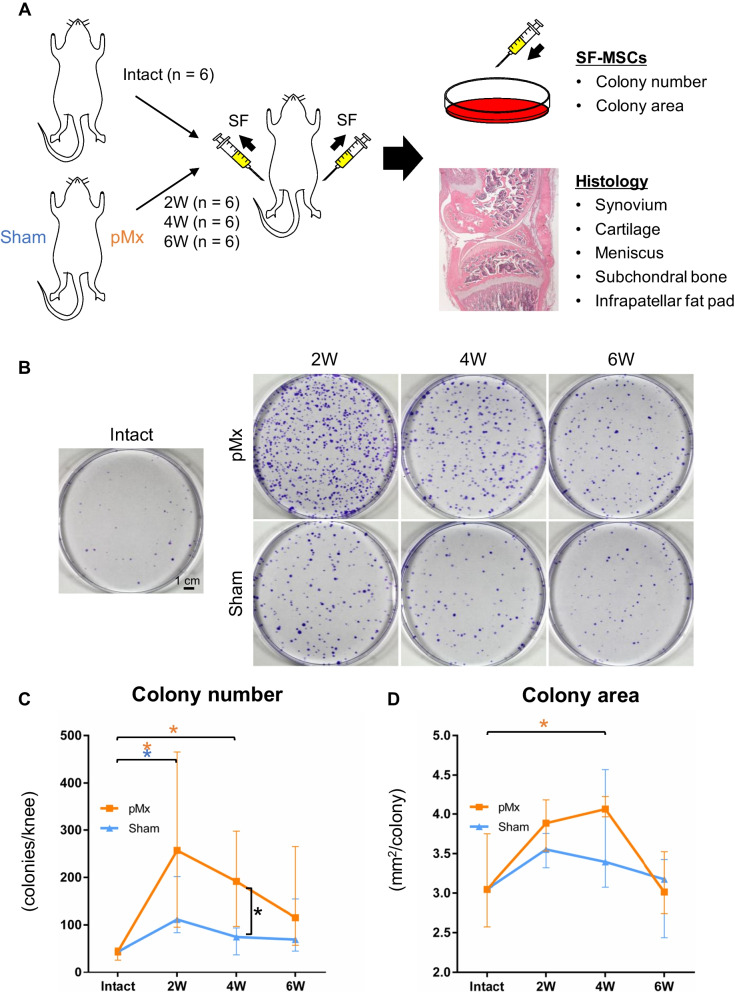
Changes in the numbers of colony-forming cells in synovial fluid (SF) and their colony areas in a rat osteoarthritis (OA) model. **A** Scheme of the experiment. Partial medial meniscectomy (pMx) was performed on either the right or left knee, and the contralateral knee received sham surgery (joint capsule incision). After 2, 4, and 6 weeks, SF and the knee joints were collected. Intact knees that received no surgery were also prepared. **B** Representative images of cell colonies stained with crystal violet. **C** Colony number per knee (*n* = 6). **D** Area per colony. *p < 0.05 by the Mann–Whitney U test between the sham and pMx groups (black asterisks). *p < 0.05 by Kruskal–Wallis/Steel tests between the intact and pMx groups (orange asterisks) and intact and sham groups (blue asterisks)

SF was collected from intact knees and from knees 2, 4, and 6 weeks after sham and pMx surgery to investigate the changes in the numbers and proliferative abilities of SF-MSCs (Fig. 1A). Colonies were formed from cells from the intact group (median 43.5 colonies/knee, IQR 29–46 colonies/knee) but more colonies were formed from cells from the pMx and sham groups (Fig. 1B and C). The colony numbers increased at 2 weeks postoperatively and then decreased over time, but were higher in the pMx group than in the sham group at each time point, especially at 4 weeks. Colony areas at 4 weeks were 1.3-fold larger in size in the pMx group than in the intact group (Fig. 1D). The sham group showed no significant changes in colony areas.

### Characteristics of colony-forming cells in the SF

**Fig. 2:**
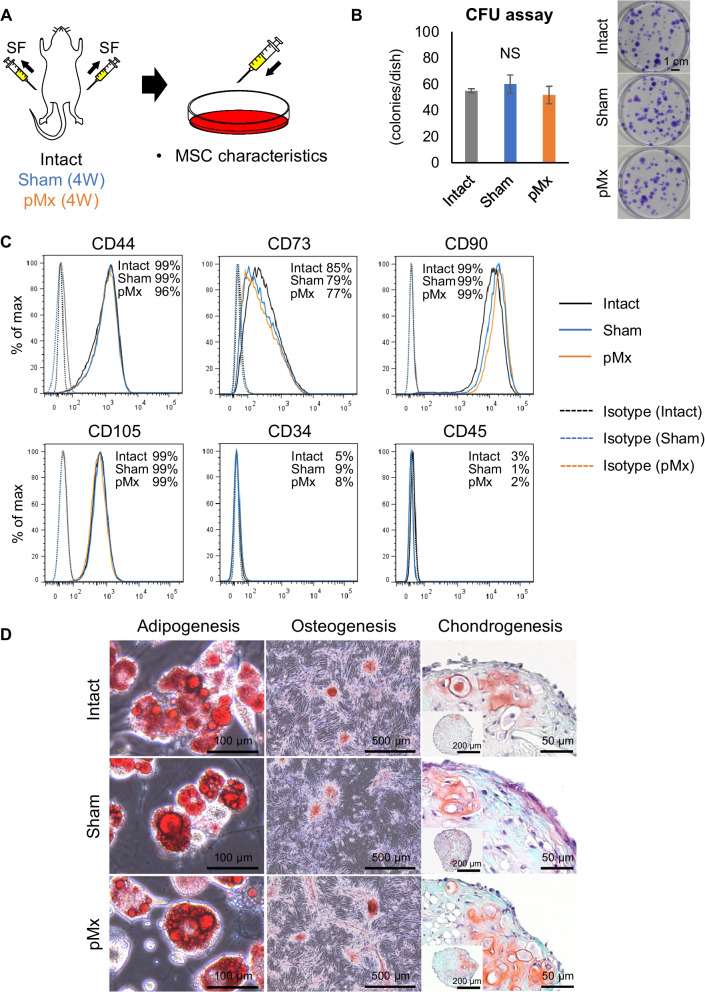
Phenotypic analysis of colony-forming cells in synovial fluid (SF). **A** Scheme of the experiment. SF was collected from intact knees and knees 4 weeks after sham and pMx surgery and cultured for 1 week. **B** Colony-forming unit (CFU) assay. The numbers of colonies and representative images of colonies stained with crystal violet are shown. **C** Representative flow cytometric histograms. **D** Differentiation assays. Representative images of oil red O, alizarin red, and safranin O staining are shown

We investigated whether the colony-forming cells in the SF possess the characteristics of MSCs by performing CFU assays, surface antigen analysis, and tri-lineage differentiation tests (Fig. 2A). The cells in all groups showed comparable colony-forming potentials (Fig. 2B), as well as similar expression patterns for surface antigens, as they were positive for CD44, CD73, CD90, and CD105 and negative for CD34 and CD45 (Fig. 2C). After adipogenic, osteogenic, and chondrogenic induction, the cells in all groups stained with oil red O, alizarin red, and safranin O, respectively (Fig. 2D). These features confirmed that the colony-forming cells in the SF had the characteristics of MSCs [6].

### Synovitis and its association with colony number and area

**Fig. 3:**
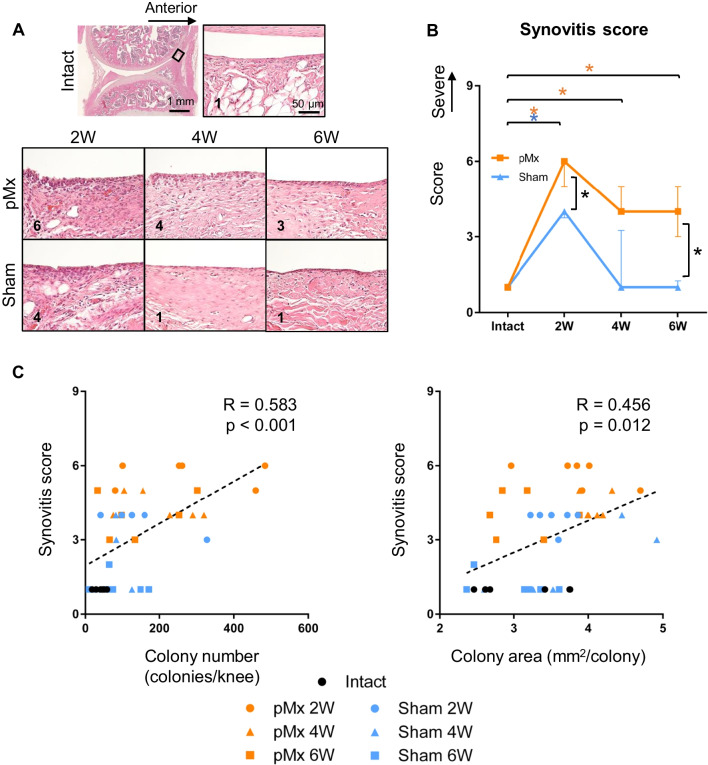
Histological evaluation of synovitis and its association with the numbers and areas of colonies. **A** Representative images of synovium stained with hematoxylin and eosin. **B** Synovitis score (*n* = 6). *p < 0.05 by the Mann–Whitney U test between sham and pMx groups (black asterisks). *p < 0.05 by Kruskal–Wallis/Steel tests between intact and pMx groups (orange asterisks) and intact and sham groups (blue asterisks). **C** Scatterplot of the synovitis score and colony numbers or colony areas. The p values were calculated using Spearman’s rank correlation and adjusted using Bonferroni’s correction method

Knee joints were evaluated histologically to elucidate which structural changes were predominantly associated with the numbers and areas of colonies. The pMx and sham groups both showed enlargement of the synovial lining layer and increased cellularity in the stroma (Fig. 3A). The synovitis score increased at 2 weeks in both groups, but it remained higher in the pMx group than in the sham group at 6 weeks (Fig. 3B). All subscores tended to be higher in the pMx group than in the sham group (Additional file 1: Fig. S1). The synovitis score was moderately correlated with the colony number (*R* = 0.583, *p* = 0.0003) and colony area (*R* = 0.456, *p* = 0.012) (Fig. 3C).

### Cartilage degeneration and its association with colony number and area

**Fig. 4:**
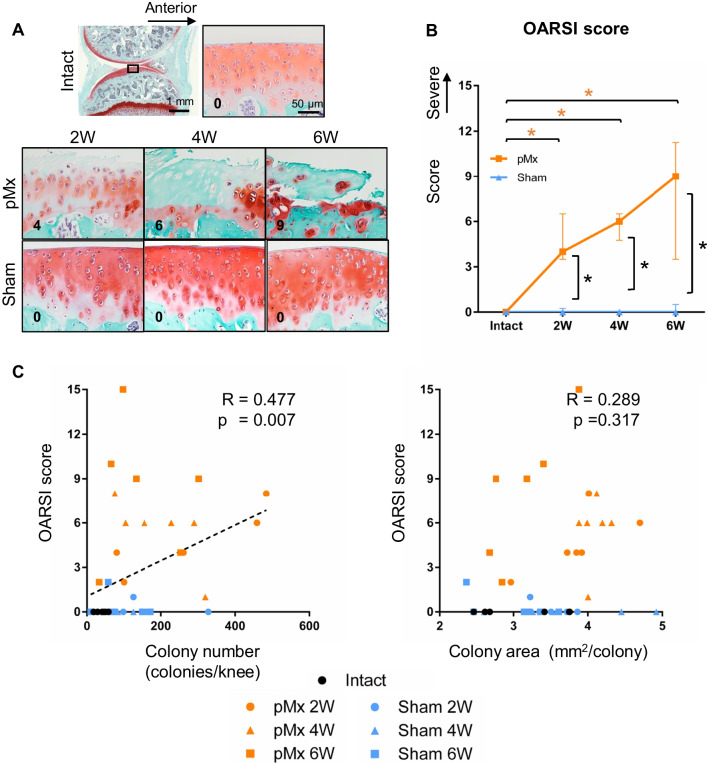
Histological evaluation of cartilage injury and its association with the numbers and areas of colonies. **A** Representative images of tibial cartilage stained with safranin O/fast green. **B** OARSI score (*n* = 6). *p < 0.05 by the Mann–Whitney U test between the sham and pMx groups (black asterisks). *p < 0.05 by Kruskal–Wallis/Steel tests between the intact and pMx groups (orange asterisks). **C** Scatterplot of OARSI scores and colony numbers or colony areas. The p values were calculated using Spearman’s rank correlation and adjusted using Bonferroni’s correction method

Safranin O staining revealed a time-dependent increase in the severity of articular cartilage lesions in the pMx group (Fig. 4A). The OARSI score increased over time in the pMx group, but remained unchanged in the sham group (Fig. 4B). The OARSI score was moderately correlated with colony number (*R* = 0.477, *p* = 0.007), but not with colony area (*R* = 0.289, *p* = 0.317) (Fig. 4C).

### Spontaneous meniscus regeneration and its association with colony number and area

**Fig. 5:**
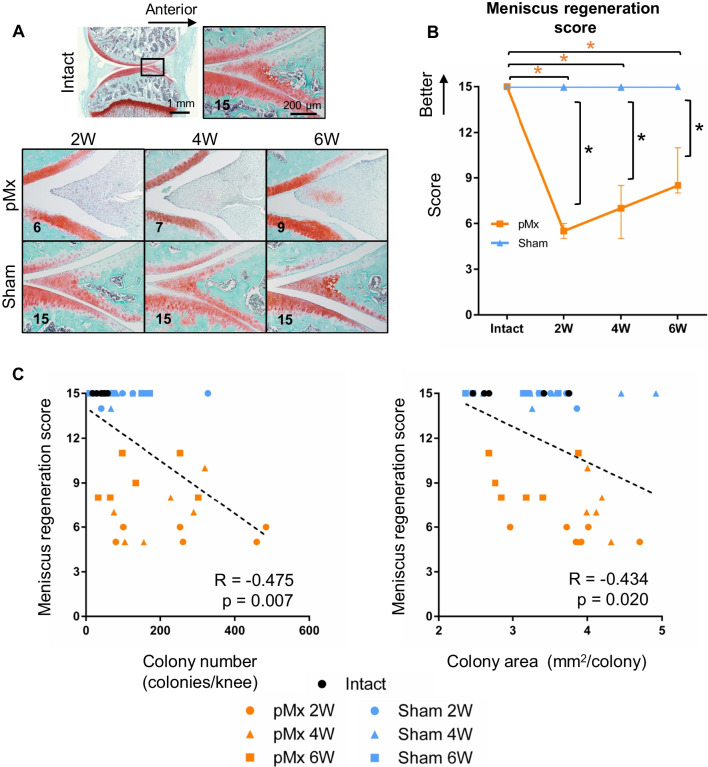
Histological evaluation of spontaneous meniscus regeneration and its association with the numbers and areas of colonies. **A** Representative images of medial meniscus stained with safranin O/fast green. **B** Meniscus regeneration score (*n* = 6). *p < 0.05 by the Mann–Whitney U test between the sham and pMx groups (black asterisks). *p < 0.05 by Kruskal–Wallis/Steel tests between the intact and pMx groups (orange asterisks). **C** Scatterplot of the meniscus regeneration scores and colony numbers or colony areas. The p values were calculated using Spearman’s rank correlation and adjusted using Bonferroni’s correction method

Fibrous tissue was formed in the meniscus resected site at 2 weeks, decreased cellularity was apparent at 4 weeks, and increased matrix staining with safranin O was evident at 6 weeks (Fig. 5A). The meniscus regeneration score substantially decreased at 2 weeks and then increased over time in the pMx group (Fig. 5B). The sham group had no meniscus damage, and therefore showed no changes in the meniscus regeneration score. The meniscus regeneration score showed a moderate negative correlation with colony number (*R* = − 0.475, *p* = 0.007) and colony area (*R* = − 0.434, *p* = 0.020) (Fig. 5C).

### BML and its association with colony number and area

The features of BML, such as bone marrow fibrosis, thickened trabeculae, and cartilage islands, were observed in the pMx group, which showed a gradual increase in BML score (Additional file 1: Fig. S2A and B). The sham group showed no changes in histology or scores. The BML score was not significantly correlated with colony number (*R* = 0.375, *p* = 0.072), and it showed no correlation with colony area (*R* = 0.18, *p* = 1) (Additional file 1: Fig. S2C).

### IFP fibrosis and its association with colony number and area

In both the sham and pMx groups, approximately 70% of the adipose tissue in the IFP was replaced by fibrous tissue at 2 weeks postoperatively and did not revert to normal levels by 6 weeks (Additional file 1: Fig. S3A and B). The IFP fibrosis rate was not significantly correlated with the colony number (*R* = 0.375, *p* = 0.073) or with the colony area (*R* = 0.201, *p* = 0.715) (Additional file 1: Fig. S3C).

### Immunohistochemical changes in the synovium and their associations with colony number and area

**Fig. 6:**
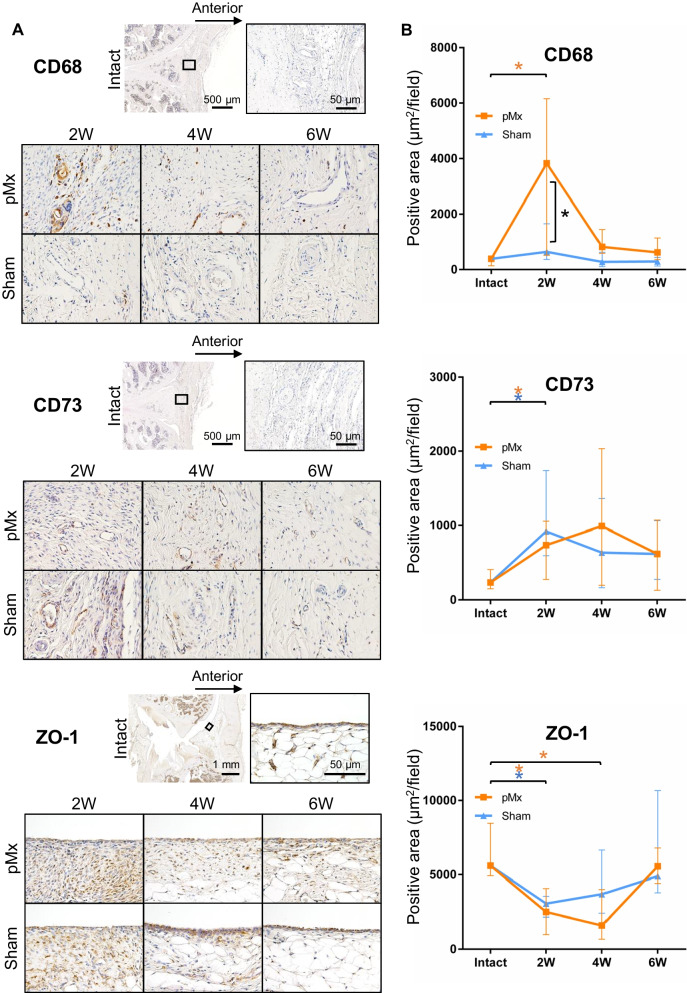
Immunohistochemical evaluation of the synovium. **A** Representative images of CD68, 73, and ZO-1. **B** Quantification of the positive areas of the immunostained cells (*n* = 6). *p < 0.05 by the Mann–Whitney U test between the sham and pMx groups (black asterisks). *p < 0.05 by Kruskal–Wallis/Steel tests between the intact and pMx groups (orange asterisks) and the intact and sham groups (blue asterisks)

The finding that synovitis was most closely associated with colony number and area prompted us to perform immunostaining for synovitis-related and MSC mobilization-related molecules (CD68, CD73, and ZO-1). CD68 is a common marker for macrophages. The CD68-positive area in the synovium did not change in the sham group, whereas it increased markedly in the pMx group at 2 weeks postoperatively (13.3-fold compared with the intact group) (Fig. 6A and B). The CD68-positive area showed a moderate correlation with colony number (*R* = 0.476, *p* = 0.001) and colony area (*R* = − 0.434, *p* = 0.020) (Additional file 1: Fig. S4A). We also performed immunostaining for CD80 (an M1 marker) and CD206 (an M2 marker) to investigate macrophage polarization [28]. The CD80-positive area did not change in either the sham or the pMx groups (Additional file 1: Fig. S5A and B). The CD206-positive area did not change in the sham group, whereas it increased in the pMx group at 2 weeks postoperatively compared with the intact group.

CD73 is a marker for MSCs; therefore, its expression in the synovium reflects the numbers of synovial MSCs [29]. CD73 was mainly expressed in the perivascular area of the synovium. The area positive for CD73 was increased in both the sham and pMx groups at 2 weeks postoperatively, but no obvious changes were detected thereafter (Fig. 6A). The CD73-positive area showed no correlation with colony number (*R* = 0.11, *p* = 0.489) (Additional file 1: Fig. S4B). Fluorescent immunostaining revealed that the CD73-positive cells were also positive for CD44 and CD271, which are markers common to MSCs (Additional file 1: Fig. S6) [30, 31]. We also used flow cytometry to determine the proportions of MSCs in synovial tissues from intact knees and from knees 4 weeks after sham or pMx surgery. The percentage of cells positive for CD73^+^CD44^+^ in CD90^+^ fibroblasts was 1.59%, 2.59%, and 4.18% in the intact, sham, and pMx groups, respectively (Additional file 1: Fig. S7).

ZO-1 is a tight junction protein that binds synovial lining macrophages on the synovial surface and functions as a barrier to regulate cell infiltration into the SF in inflammatory joints [26]. ZO-1 was continuously distributed in the synovial lining layer of intact knees (Fig. 6A). In the pMx group, ZO-1 expression in the lining weakened steadily until 4 weeks postoperatively (Fig. 6B). For the sham group, ZO-1 expression decreased at 2 weeks but returned to the normal level at 6 weeks. The ZO-1 positive area was not significantly correlated with colony number (*R* = 0.253, *p* = 0.106) (Additional file 1: Fig. S4B).

### Gene expression profiling of colony-forming cells in SF

**Fig. 7:**
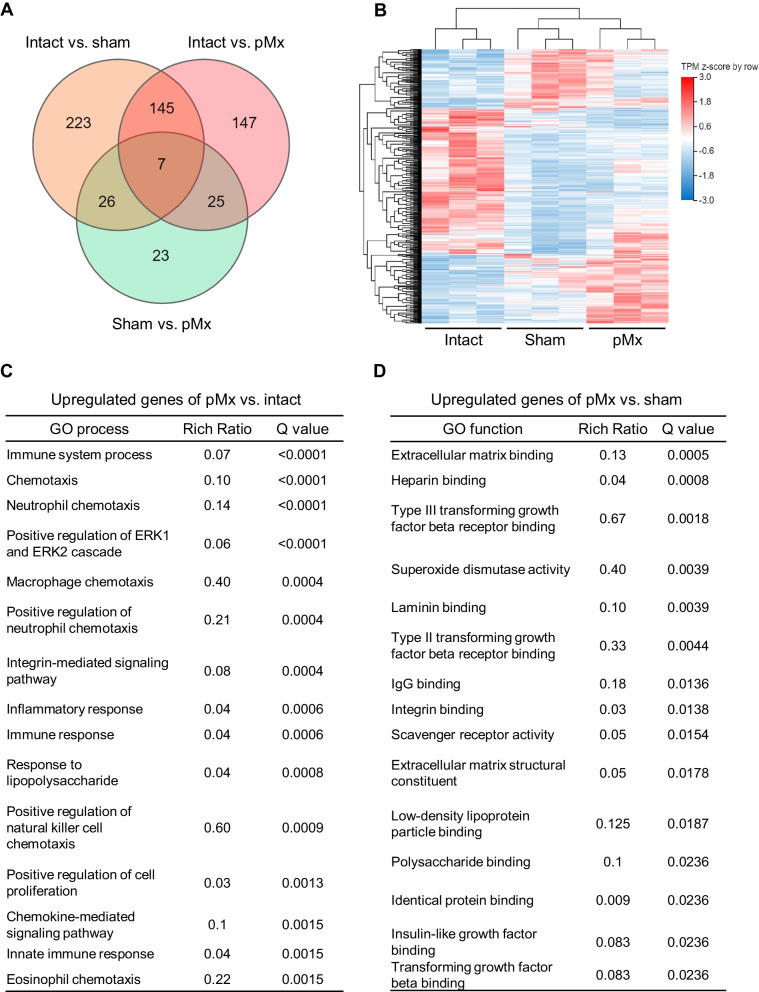
RNA sequencing of colony-forming cells in synovial fluid. **A** Venn diagrams of differentially expressed genes (DEGs) among the intact, sham, and pMx groups. **B** Hierarchical clustering heatmap of DEGs (*n* = 3). **C** The top 15 gene ontology (GO) biological processes upregulated in the pMx group compared with the intact group. **D** The top 15 GO molecular function upregulated in the pMx group compared with the sham group

RNA sequencing was performed to assess qualitative changes in colony-forming cells in SF from intact, sham, and pMx knees. Analysis of the DEGs identified 401 genes (up 146, down 255) for sham vs. intact knees, 324 genes (up 189, down 135) for pMx vs. intact knees, and 81 genes (up 73, down 8) for pMx vs. sham knees (Fig. 7A). Heatmap cluster analysis of DEGs showed a clear segregation of the intact, sham, and pMx groups (Fig. 7B).

GO enrichment analysis of the biological processes of the DEGs that were highly expressed in the pMx group compared to the intact group identified 135 terms with q-values < 0.05. The top 15 terms included “positive regulation of ERK1 and ERK2 cascade” and “positive regulation of cell proliferation” (Fig. 7C). These terms included 26 cell proliferation-related genes, with Wnt10b showing the most markedly increased expression (Additional file 1: Fig. S8). The top 15 terms also included inflammatory response-related terms (“immune system process,” “chemotaxis,” “neutrophil chemotaxis,” “macrophage chemotaxis,” “positive regulation of neutrophil chemotaxis,” “immune response,” “positive regulation of natural killer cell chemotaxis,” and “eosinophil chemotaxis”), with 35 genes annotated (Additional file 1: Fig. S8). GO enrichment analysis of the biological processes of the DEGs that were more highly expressed in the sham group than in the intact group identified 126 terms with q-values < 0.05. The top 15 terms included “cell adhesion,” “angiogenesis,” and “cell migration” (Additional file 1: Fig. S9).

**Fig. 8:**
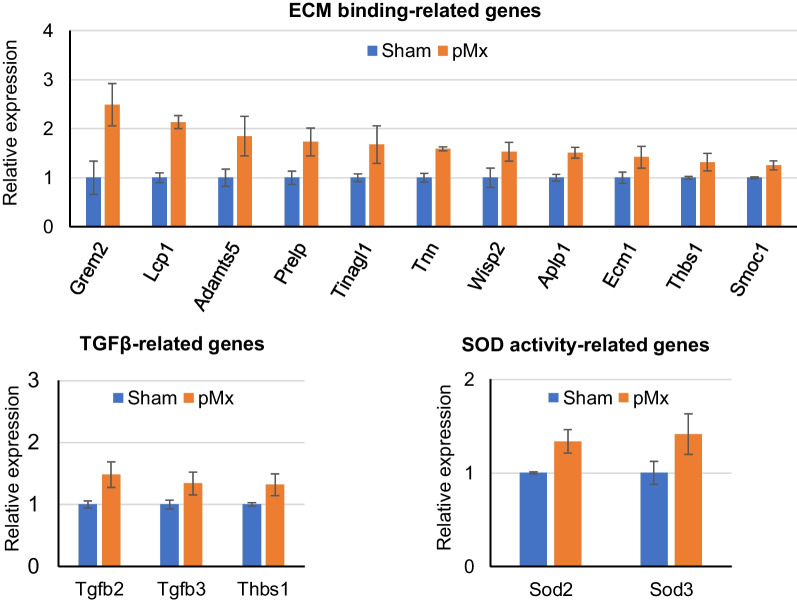
Expression patterns of genes associated with extracellular matrix (ECM) biding-related genes, TGFβ-related genes, and superoxide dismutase activity (SOD) activity-related genes in the sham and pMx groups

GO enrichment analysis of the molecular functions of the DEGs that were highly expressed in the pMx group compared to the sham group identified 17 terms with q-values < 0.05. The top 15 terms included many ECM binding-related terms (“extracellular matrix binding,” “heparin binding,” “laminin binding,” and “integrin binding”). In these terms, 11 genes, including Grem2, Lcp1, Adamts5, and Prelp, were upregulated (Fig. 8). We also found TGFβ-related terms (“type III transforming growth factor beta receptor binding,” “type II transforming growth factor beta receptor binding,” and “transforming growth factor beta binding”) and “superoxide dismutase (SOD) activity” on the list. Tgfb2, Tgfb3, and Thbs1 were upregulated in the TGFβ-related terms, and Sod2 and Sod3 upregulated in the “superoxide dismutase (SOD) activity” group.

## Discussion

SF-MSCs are considered to play a significant role in the endogenous repair of intra-articular tissues. An understanding of how SF-MSCs in OA knee joints change their numbers and phenotypes is critical for developing OA treatments that do not require the application of exogenous MSCs. The findings of this study clarified the changes in SF-MSC numbers during OA progression in rats and indicated that these changes were correlated with cartilage degeneration and meniscus regeneration, especially in cases of severe synovitis. Immunostaining of the synovium revealed a correlation between the areas positive for CD68 (a macrophage marker) and the changes in the number and colony area of SF-MSCs. The expression of proliferation-related genes was higher in SF-MSCs from OA knee joints than from intact knees. The expression of genes related to ECM binding, TGF-β, and SOD activity was also higher in SF-MSCs from OA knees than from sham knees.

In this study, we first used a rat OA model to investigate the time-course changes in the numbers of colony-forming cells in SF and the colony areas formed by these cells in culture. The SF from the intact control group contained a few colony-forming cells. By contrast, the number of colonies increased greatly in the sham and pMx groups 2 weeks after surgery; this could be a response to the surgical invasiveness. Our previous study demonstrated a similar result, as the numbers of SF-MSCs increased after arthroscopic operation in knees with degenerative meniscus injury [10]. The colony areas at 4 weeks were 1.3-fold larger for the pMx group than for the intact group. Since the colony area reflects the cell number, this greater area indicated an upregulation of the proliferation ability of SF-MSCs in an OA environment.

Histological evaluation of the rat knee joints revealed that synovitis was most strongly correlated with colony number. This finding was consistent with our previous findings that inflammatory cytokines enhanced the migration ability of synovial MSCs and that synovium-resident MSCs appeared to mobilize into the SF in response to synovitis [21]. Immunostaining of the synovium to identify events responsible for changes in SF-MSCs demonstrated a significant correlation between the CD68-positive area in the synovium and the SF-MSC colony number and area.

Recent research has begun to clarify the crosstalk between MSCs and macrophages [32]. Macrophages are known to secrete high levels of several growth factors that can promote the migration and proliferation of synovial MSCs [21, 33, 34]. Our findings suggest that synovial macrophages regulate the mobilization of synovial MSCs into the SF in OA. However, the specific proportion of cells in the synovial tissue was not measured in the present study. Future studies should include flow cytometry examinations to establish the proportions and interactions of synovial cells [24].

The area positive for CD73, a marker of MSCs, increased after both sham and pMx surgery, but showed no correlation with colony number. This indicates that increasing the numbers of synovial MSCs, which are viewed as a potential source of SF-MSCs, does not lead to increases in SF-MSC numbers. Therefore, eliciting mobilization is more important than eliciting the proliferation of synovial MSCs to increase the number of SF-MSCs and promote endogenous tissue regeneration.

The number of colonies was moderately correlated with cartilage damage and meniscal regeneration. We have previously reported that cartilage and meniscal injuries are associated with increased colony-forming cell numbers in the SF [13, 14], in agreement with the present results. The injured meniscus and cartilage in OA can release many cytokines, such as IGF-1, PDGFs, and MCP-1, which reportedly mobilize and propagate MSCs [35–39]. We also showed that BML and IFP fibrosis are weakly correlated with colony number. Although bone marrow and IFP are possible sources of SF-MSCs [40, 41], the present results suggest that the origin of the SF-MSCs is more likely the synovium.

We performed RNA-seq at 4 weeks postoperatively to evaluate the phenotypic changes of SF-MSCs in OA. Gene ontology enrichment analysis demonstrated higher expression of cell proliferation-related genes, particularly Wnt10b, in the pMx group than in the intact group. Liu et al. reported that the overexpression of Wnt10b in bone marrow MSCs results in high proliferative ability [42]. The increased colony area seen for the pMx group at 4 weeks, taken together with the increase in proliferation-related genes, suggests that the proliferative capacity is higher for SF-MSCs in OA knees than in intact knees.

In addition, many terms related to the inflammatory response were detected. MSCs are highly responsive to inflammation in vivo and produce many soluble factors that regulate the immune response [37]. Upregulation of the expression of inflammatory response-related genes in the pMx group is probably a consequence of the synovitis that was present at 4 weeks postoperatively.

Comparison of the enrichment analysis results between the pMx and sham groups identified genes related to ECM binding, TGFβ signaling, and SOD activity. The ECM binding-related genes included regulators of ECM production and degradation, attachment to ECM, and cartilage development, suggesting that SF-MSCs in the pMx group may have a greater ability to adhere to damaged cartilage/meniscus and to promote ECM remodeling compared with the SF-MSCs in the sham group. Among these enriched genes, Grem2, encoding an antagonist of bone morphogenic protein (BMP), showed the highest expression in the pMx group. Wang et al. showed that human bone marrow MSCs that overexpress Grem2 had poor in vitro osteogenic ability, and that suppression of Grem2 resulted in increased in vivo bone formation [43]. Thus, a higher expression of Grem2 in SF-MSCs may suppress their own ossification and that of the surrounding joint tissue.

TGFβ signaling is essential for chondrogenesis of MSCs and chondrocytes [44, 45], and extracellular SOD3 is reported to suppress chondrocyte apoptosis and ECM destruction induced by oxidative stress [46, 47]. Collectively, these data indicate that the tissue-reparative properties are greater for SF-MSCs from OA knees than from knees with no structural joint damage.

The present finding that SF-MSCs showed tissue-reparative phenotypes even in an OA environment supports the investigation of therapeutic strategies that could recruit endogenous synovial MSCs into the SF to promote joint regeneration. However, the numbers of SF-MSCs in human knees are much lower than the numbers of synovial MSCs required for transplantation [10]. The numbers of endogenous MSCs will need to be increased to achieve endogenous tissue regeneration. We previously demonstrated that intra-articular injection of PDGF-BB effectively mobilized synovial MSCs into the SF [21]. Baboolal et al. reported a mechanical method to increase SF-MSCs that involved agitation of the synovium during arthroscopy [48].

One major limitation of our study is that we used culture-expanded MSCs for RNA sequencing because uncultured SF-MSCs are rare and difficult to collect, especially in intact knees. However, in vitro culture is known to affect gene expression profiles [49]. Single-cell RNA sequencing, which enables transcriptome analysis of rare cell populations at a high resolution, will be a promising solution [50]. Another limitation is that we adopted a surgical OA induction method. Since the joint capsule incision itself can cause strong synovitis, our surgical OA model may not have reflected natural OA progression. Less invasive models, such as noninvasive anterior cruciate ligament rupture and intra-articular fracture models, should be explored [51, 52].

## Conclusions

This study revealed that the number of SF-MSCs was most closely correlated with the severity of synovitis in a rat OA model. The expression of tissue-reparative genes was greater in SF-MSCs from OA knees than in SF-MSCs from knees with no structural joint damage.

### Supplementary Information

**Additional file 1.** **Fig. S1 **Synovitis subscores. **Fig. S2 **Histological evaluation of a bone marrow lesion (BML) and its association with the numbers and areas of colonies. **(A) **Representative images of subchondral bone stained with hematoxylin and eosin. **(B)** BML score (n = 6). *p < 0.05 by the Mann–Whitney U test between the sham and pMx groups (black asterisks). *p < 0.05 by Kruskal–Wallis/Steel tests between the intact and pMx groups (orange asterisks). **(C)** Scatterplot of the BML scores and colony numbers or colony areas. The p values were calculated using Spearman’s rank correlation and adjusted using Bonferroni’s correction method. **Fig. S3 **Histological evaluation of the infrapatellar fat pad (IFP) fibrosis area and its association with the numbers and areas of colonies. **(A) **Representative images of IFP stained with hematoxylin and eosin. **(B)** IFP fibrosis area (n = 6). *p < 0.05 by the Mann–Whitney U test between the sham and pMx groups (black asterisks). *p < 0.05 by Kruskal–Wallis/Steel tests between the intact and pMx groups (orange asterisks). **(C)** Scatterplot of the BML scores and colony numbers or colony areas. The p values were calculated using Spearman’s rank correlation and adjusted using Bonferroni’s correction method. **Fig. S4 **Association of immunostaining quantification of synovium with the numbers and areas of colonies.** (A) **Scatterplot of the CD68-positive areas in synovial tissues and colony numbers or colony areas. **(B) **Scatterplot of the CD73 and ZO-1 positive areas in synovial tissues and colony numbers. **Fig. S5 **Immunohistochemical evaluation of the synovium. (A) Representative images of CD80 and CD206 staining. (B) Quantification of the positive areas of the immunostained cells (n = 6). *p < 0.05 by Kruskal–Wallis/Steel tests between the intact and pMx groups (orange asterisks). **Fig. S6 **Immunofluorescence of the synovium. (A) Representative images of negative control tissues stained with Alexa488 (green), Alexa555 (red), and DAPI (blue). (B) Representative images of CD44 (green) and CD73 (red) staining. (C) Representative images of CD271 (green) and CD73 (red) staining. **Fig. S7 **Flow cytometry analysis of synovial tissues from intact knees and from knees 4 weeks after sham or pMx surgery. **Fig. S8 **Expression patterns of genes associated with cell proliferation and the inflammatory response in the intact and pMx groups. **Fig. S9 **The top 15 gene ontology (GO) biological processes showing greater upregulation in the sham group than in the intact group.

**Additional file 2. Table S1** Histological scoring system for synovitis (Krenn’s score). **Table S2** Histological scoring system for cartilage degeneration (OARSI score). **Table S3** Histological scoring system for meniscus regeneration (modified Pauli’s score). **Table S4** Histological scoring system for bone marrow lesion (osteoarthritis bone score).

## Data Availability

The datasets generated and analyzed during the current study are available from the corresponding author on reasonable request. All RNA sequencing datasets were deposited in the National Center for Biotechnology Information’s Gene Expression Omnibus with accession number GSE227922.

## Abbreviations

α-MEM: α-Modified essential medium;
BMLs: Bone marrow lesions
BMP: Bone morphogenetic protein
DAB: Diaminobenzidine
DEGs: Differentially expressed genes
DMSO: Dimethyl sulfoxide
ECM: Extracellular matrix
EDTA: Ethylenediaminetetraacetic acid
FBS: Fetal bovine serum
HE: Hematoxylin and eosin
IFP: Infrapatellar fat pad
ITS: Insulin–transferrin–selenium
MSCs: Mesenchymal stem cells
OA: Osteoarthritis
OABS: Osteoarthritis bone score
PBS: Phosphate buffered saline
pMx: Partial medial meniscectomy
SF: Synovial fluid
SOD: Superoxide dismutase
TBS-T: Tris-buffered saline containing 0.1% Tween-20
TPM: Transcripts per million
