# Supplementary material for: Mesenchymal stem cells in synovial fluid increase in number in response to synovitis and display more tissue-reparative phenotypes in osteoarthritis
Source: Stem Cell Res Ther. 2023 Sep 8;14:244. doi: 10.1186/s13287-023-03487-1 (PMC10485949; doi:10.1186/s13287-023-03487-1)
Supplement: Supplementary file 1 — Additional file 1. Fig. S1 Synovitis subscores. Fig. S2 Histological evaluation of a bone marrow lesion (BML) and its association with the numbers and areas of colonies. (A) Representative images of subchondral bone stained with hematoxylin and eosin. (B) BML score (n = 6). *p < 0.05 by the Mann–Whitney U test between the sham and pMx groups (black asterisks). *p < 0.05 by Kruskal–Wallis/Steel tests between the intact and pMx groups (orange asterisks). (C) Scatterplot of the BML scores and colony numbers or colony areas. The p values were calculated using Spearman’s rank correlation and adjusted using Bonferroni’s correction method. Fig. S3 Histological evaluation of the infrapatellar fat pad (IFP) fibrosis area and its association with the numbers and areas of colonies. (A) Representative images of IFP stained with hematoxylin and eosin. (B) IFP fibrosis area (n = 6). *p < 0.05 by the Mann–Whitney U test between the sham and pMx groups (black asterisks). *p < 0.05 by Kruskal–Wallis/Steel tests between the intact and pMx groups (orange asterisks). (C) Scatterplot of the BML scores and colony numbers or colony areas. The p values were calculated using Spearman’s rank correlation and adjusted using Bonferroni’s correction method. Fig. S4 Association of immunostaining quantification of synovium with the numbers and areas of colonies. (A) Scatterplot of the CD68-positive areas in synovial tissues and colony numbers or colony areas. (B) Scatterplot of the CD73 and ZO-1 positive areas in synovial tissues and colony numbers. Fig. S5 Immunohistochemical evaluation of the synovium. (A) Representative images of CD80 and CD206 staining. (B) Quantification of the positive areas of the immunostained cells (n = 6). *p < 0.05 by Kruskal–Wallis/Steel tests between the intact and pMx groups (orange asterisks). Fig. S6 Immunofluorescence of the synovium. (A) Representative images of negative control tissues stained with Alexa488 (green), Alexa555 (red), and DAPI (blue). (B) Represent [file 13287_2023_3487_MOESM1_ESM.pdf]

Fig. S1

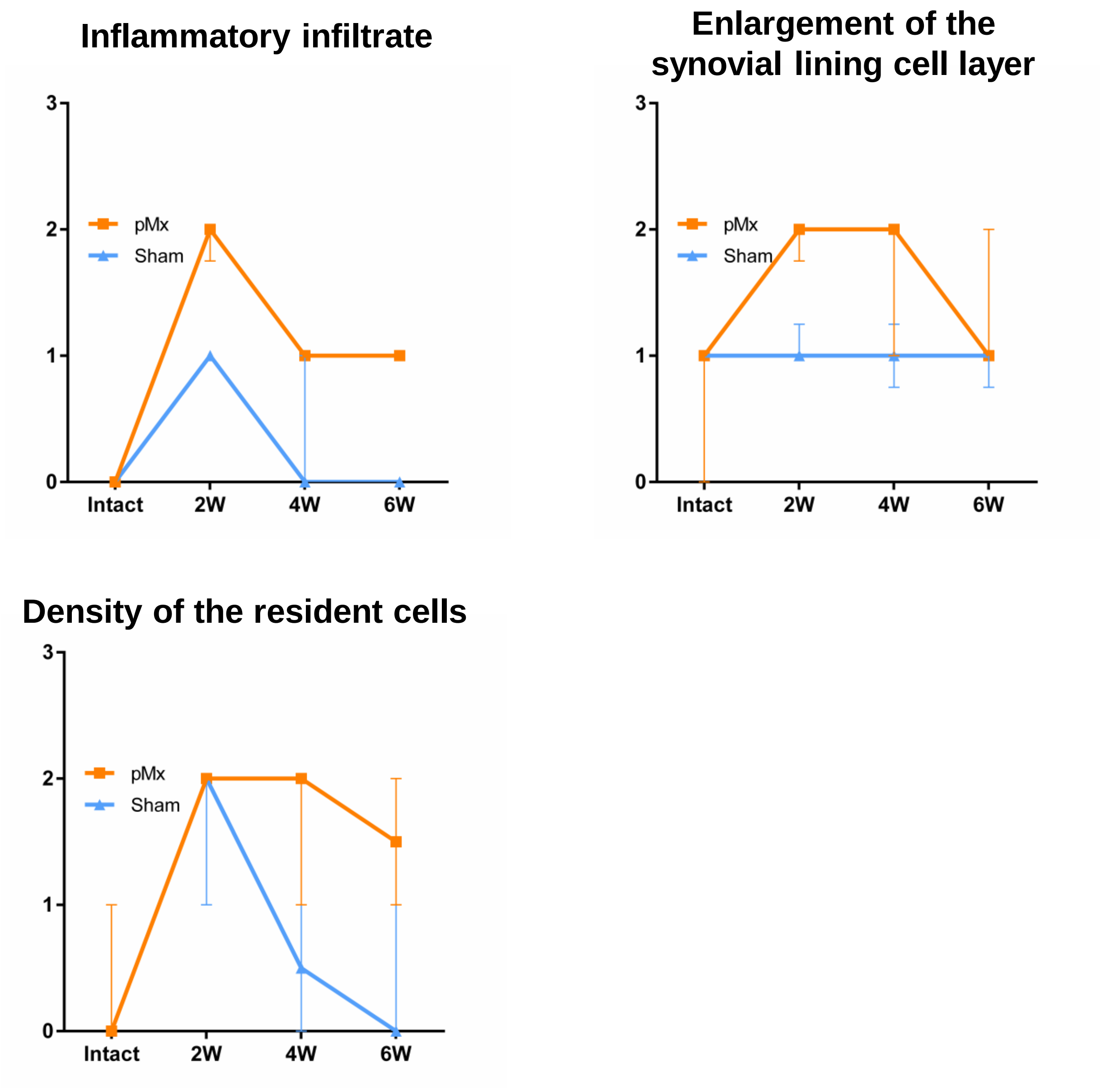

Inflammatory infiltrate
3
2
1
0
pMx
Sham
Intact
2W
4W
6W
Enlargement of the synovial lining cell layer
3
2
1
0
pMx
Sham
Intact
2W
4W
6W
Density of the resident cells
3
2
1
0
pMx
Sham
Intact
2W
4W
6W

Fig. S2

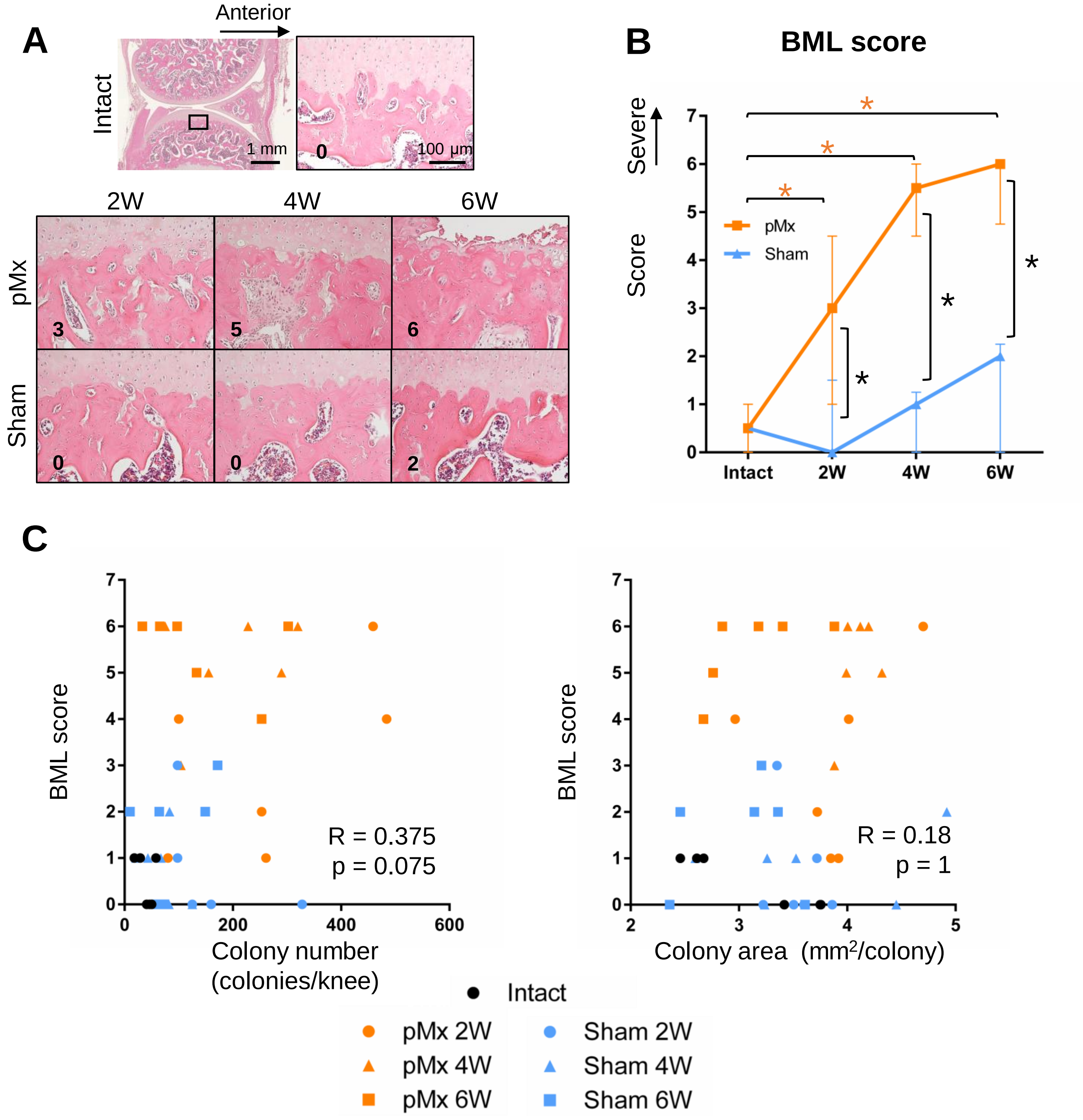

A
Anterior
Intact
1 mm
0
100 µm
2W
4W
6W
pMx
Sham
3
5
6
0
0
2
B
BML score
Severe
Score
pMx
Sham
*
Intact
2W
4W
6W
C
BML score
R = 0.375
p = 0.075
Colony number
(colonies/knee)
BML score
R = 0.18
p = 1
Colony area (mm²/colony)
Intact
pMx 2W
pMx 4W
pMx 6W
Sham 2W
Sham 4W
Sham 6W

Fig. S3

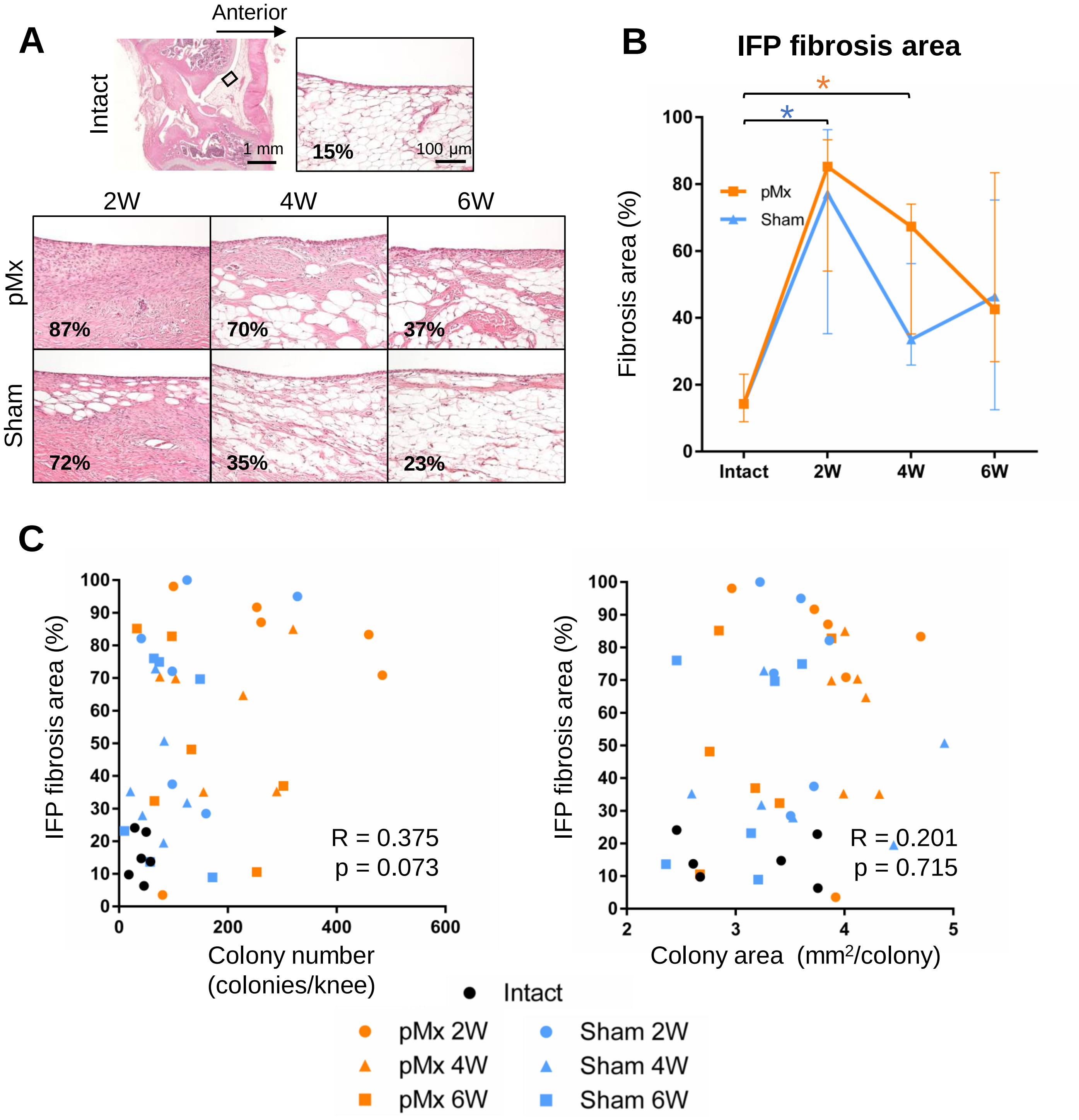
A
Anterior
Intact
1 mm
15%
100 µm
2W
4W
6W
pMx
Sham
87%
70%
37%
72%
35%
23%
B
IFP fibrosis area
Fibrosis area (%)
pMx
Sham
Intact
2W
4W
6W
C
IFP fibrosis area (%)
Colony number
(colonies/knee)
R = 0.375
p = 0.073
Colony area (mm²/colony)
R = 0.201
p = 0.715
Intact
pMx 2W
pMx 4W
pMx 6W
Sham 2W
Sham 4W
Sham 6W

Fig. S4

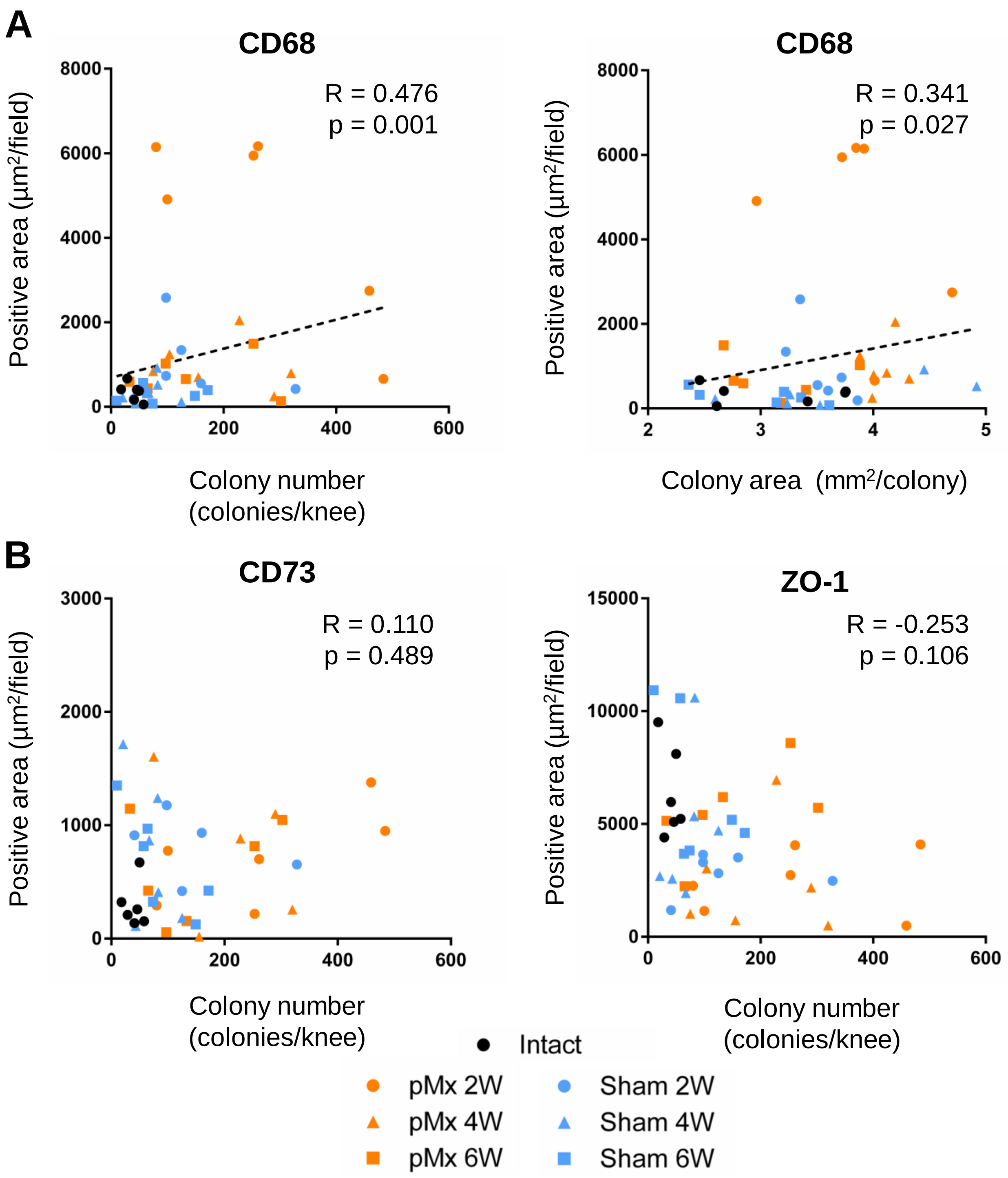

A
CD68
R = 0.476
p = 0.001
Positive area (µm2/field)
Colony number
(colonies/knee)
CD68
R = 0.341
p = 0.027
Positive area (µm2/field)
Colony area (mm2/colony)
B
CD73
R = 0.110
p = 0.489
Positive area (µm2/field)
Colony number
(colonies/knee)
ZO-1
R = -0.253
p = 0.106
Positive area (µm2/field)
Colony number
(colonies/knee)
Intact
pMx 2W
pMx 4W
pMx 6W
Sham 2W
Sham 4W
Sham 6W

Fig. S5

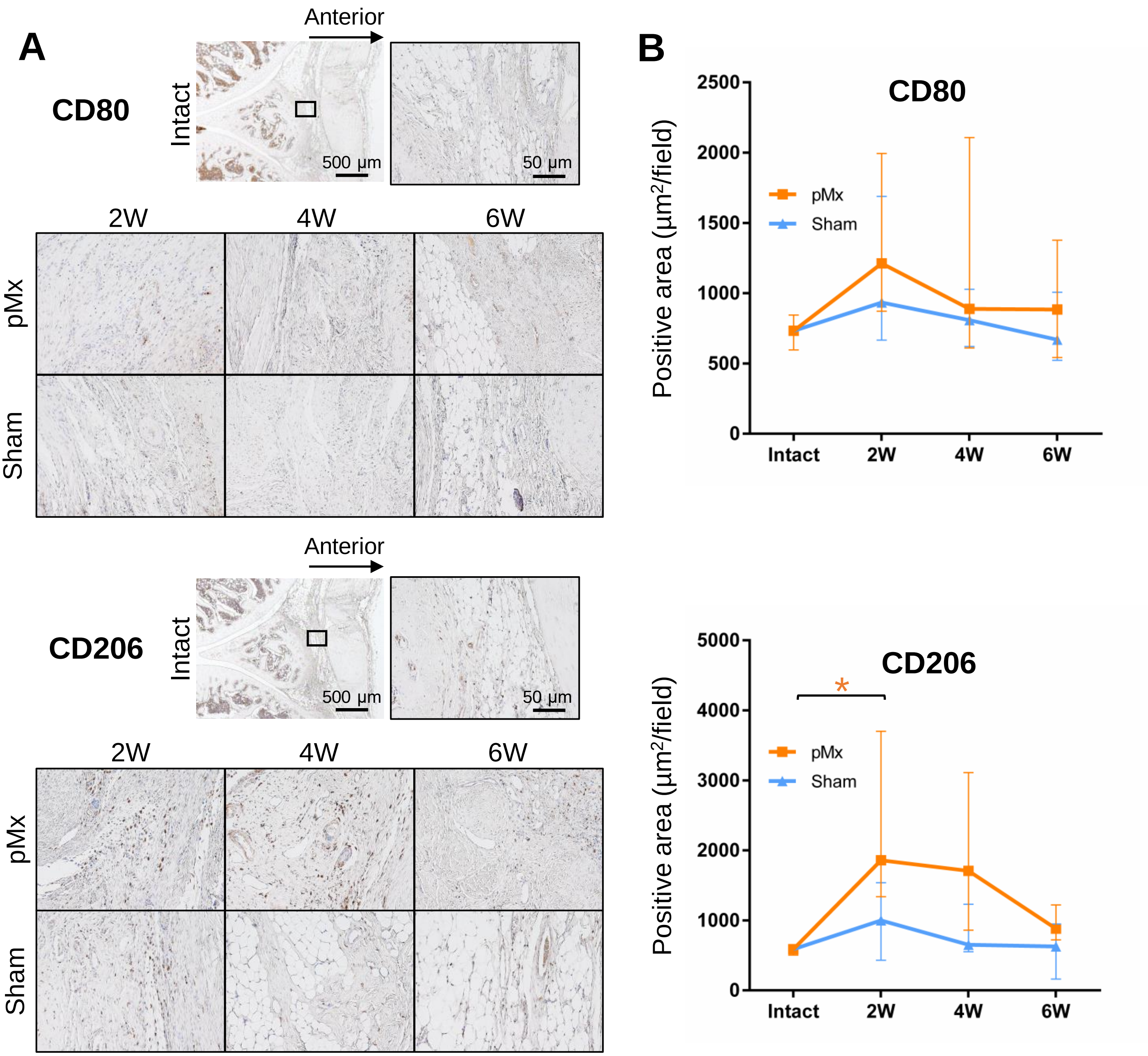

A
CD80
Intact
Anterior
500 µm
50 µm
2W
4W
6W
pMx
Sham
CD206
Intact
Anterior
500 µm
50 µm
2W
4W
6W
pMx
Sham
B
CD80
Positive area (µm2/field)
2500
2000
1500
1000
500
0
pMx
Sham
Intact
2W
4W
6W
CD206
*
Positive area (µm2/field)
5000
4000
3000
2000
1000
0
pMx
Sham
Intact
2W
4W
6W

Fig. S6

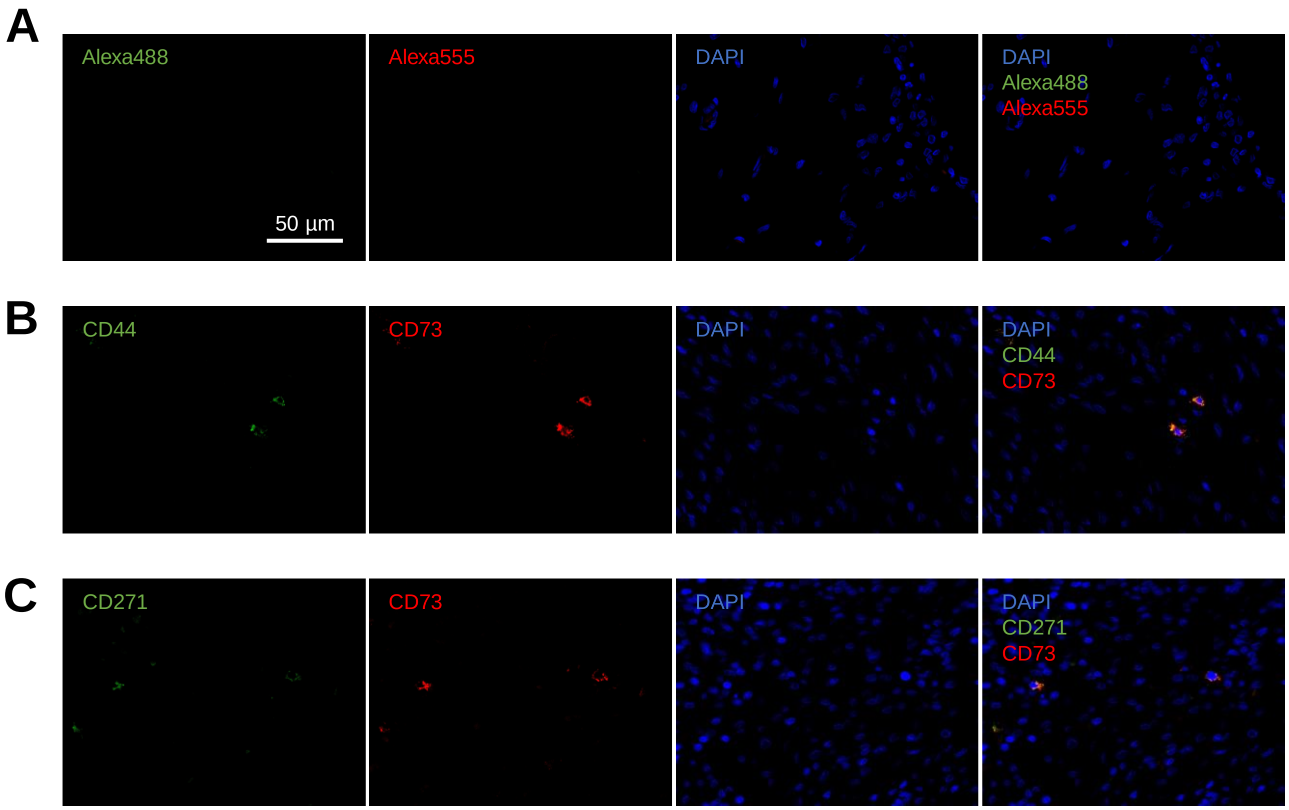
A
Alexa488
50 µm
Alexa555
DAPI
DAPI
Alexa488
Alexa555
B
CD44
CD73
DAPI
DAPI
CD44
CD73
C
CD271
CD73
DAPI
DAPI
CD271
CD73

Fig. S7

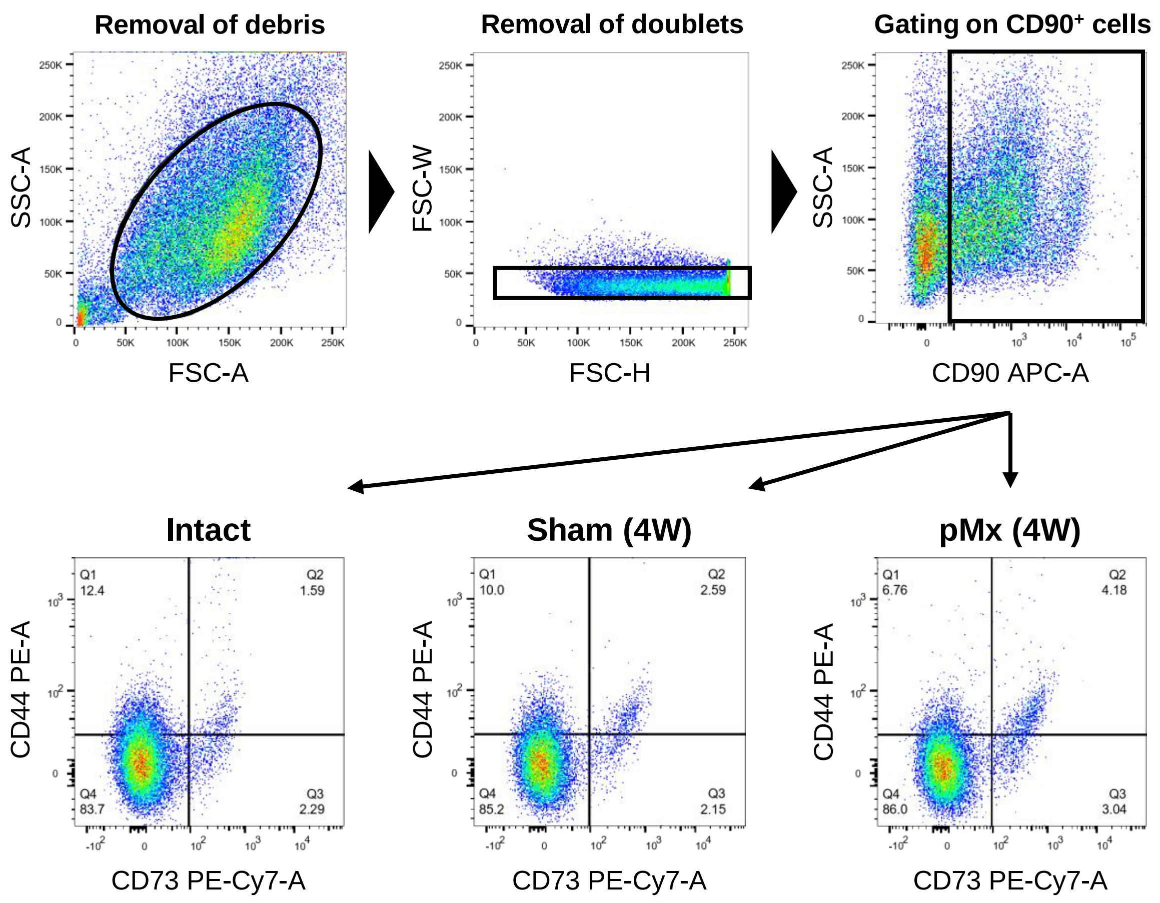

Removal of debris
Removal of doublets
Gating on CD90+ cells
SSC-A
FSC-A
FSC-W
FSC-H
SSC-A
CD90 APC-A
Intact
Sham (4W)
pMx (4W)
CD44 PE-A
CD73 PE-Cy7-A
Q1 12.4
Q2 1.59
Q4 83.7
Q3 2.29
Q1 10.0
Q2 2.59
Q4 85.2
Q3 2.15
Q1 6.76
Q2 4.18
Q4 86.0
Q3 3.04

Fig. S8

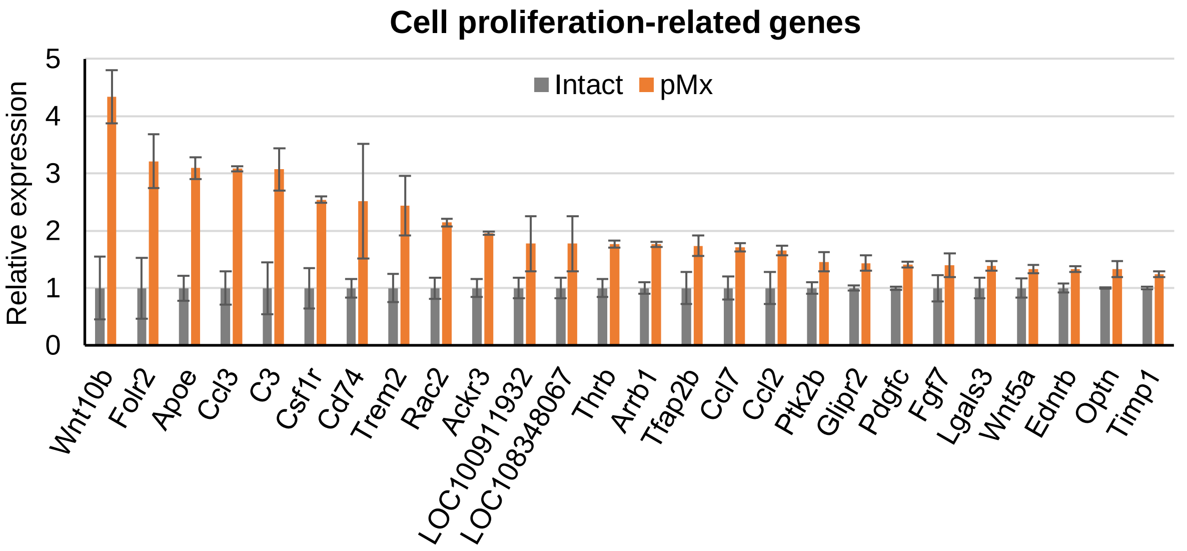
Cell proliferation-related genes
Relative expression
0
1
2
3
4
5
Intact
pMx
Wnt10b
Folr2
Apoe
Ccl3
C3
Csf1r
Cd74
Trem2
Rac2
Ackr3
LOC100911932
LOC108348067
Thrb
Arrb1
Tfap2b
Ccl7
Ccl2
Ptk2b
Glipr2
Pdgfc
Fgf7
Lgals3
Wnt5a
Ednrb
Optn
Timp1

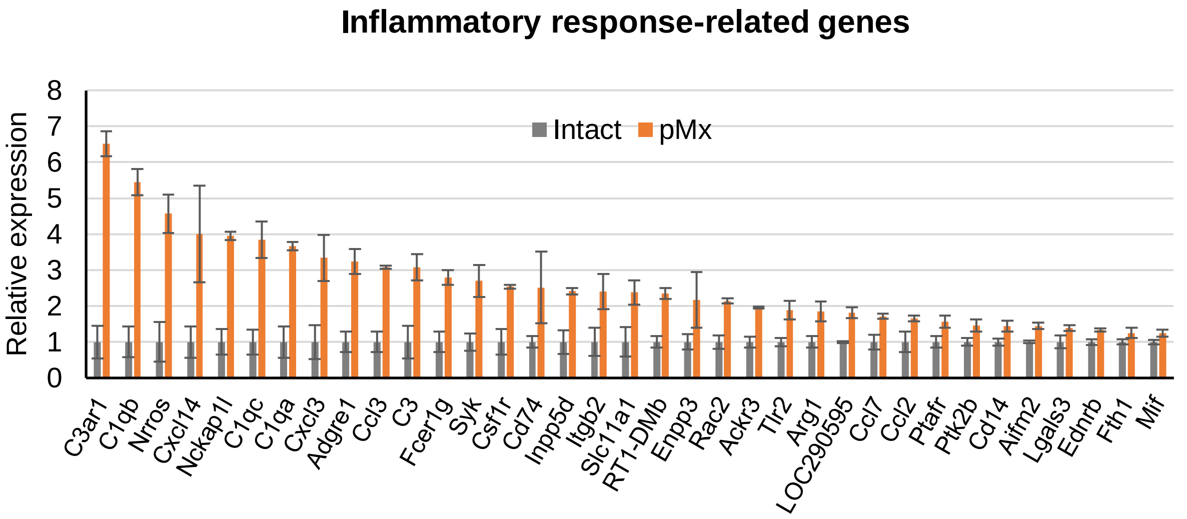
Inflammatory response-related genes
Relative expression
0
1
2
3
4
5
6
7
8
Intact
pMx
C3ar1
C1qb
Nrros
Cxcl14
Nckap1l
C1qc
C1qa
Cxcl3
Adgre1
Ccl3
C3
Fcer1g
Syk
Csf1r
Cd74
Inpp5d
Itgb2
Slc11a1
RT1-DMb
Enpp3
Rac2
Ackr3
Tlr2
Arg1
LOC290595
Ccl7
Ccl2
Ptafr
Ptk2b
Cd14
Aifm2
Lgals3
Ednrb
Fth1
Mif

Fig. S9

Upregulated genes of sham vs. intact

GO process	Rich Ratio	Q value
Cell adhesion	0.08	<0.0001
Angiogenesis	0.11	<0.0001
Positive regulation of Endothelial cell migration	0.21	<0.0001
Response to mechanical stimulus	0.12	<0.0001
Multicellular organism development	0.05	0.0002
Mesodermal cell differentiation	0.45	0.0004
Integrin-mediated signaling pathway	0.12	0.0005
Retina vasculature morphogenesis in camera-type eye	0.57	0.0012
Positive regulation of peptidyl-tyrosine phosphorylation	0.11	0.0022
Cell migration	0.07	0.0030
Extracellular matrix organization	0.09	0.0031
Negative regulation of cell proliferation	0.05	0.0038
Wound healing	0.09	0.0042
Positive regulation of ovulation	0.75	0.0042
Positive regulation of ERK1 and ERK2 cascade	0.06	0.0042
